# Emerging Techniques of Translational Research in Immuno-Oncology: A Focus on Non-Small Cell Lung Cancer

**DOI:** 10.3390/cancers17132244

**Published:** 2025-07-04

**Authors:** Mora Guardamagna, Eduardo Zamorano, Victor Albarrán-Artahona, Andres Mesas, Jose Carlos Benitez

**Affiliations:** 1 Department of Cancer Medicine, Gustave Roussy, Paris-Saclay University, 94805 Villejuif, France; mora.guardamagna@gustaveroussy.fr (M.G.);; 2 Thoracic Tumors Unit, Medical Oncology Department, Virgen de la Victoria University Hospital, IBIMA, 29010 Málaga, Spain; 3 Biomedical Research Institute of Malaga, 29590 Malaga, Spain

**Keywords:** non-small cell lung cancer (NSCLC), immune checkpoint blockers (ICB), artificial intelligence, translational medicine, mechanisms of resistance, liquid biopsy, immune biomarkers

## Abstract

The advent of personalized medicine and novel therapeutic strategies has transformed the treatment landscape of non-small cell lung cancer (NSCLC), significantly improving patient survival. However, only a minority of patients experience a durable benefit, as intrinsic or acquired resistance remains a major challenge. Understanding the complex mechanisms of resistance—linked to tumor biology, the tumor microenvironment (TME), and host factors—is crucial to overcoming these barriers. Recent innovations in diagnostics, including artificial intelligence and liquid biopsy, offer promising tools to refine therapeutic decisions. Machine Learning and Deep Learning provide predictive algorithms that enhance diagnostic accuracy and prognostic assessment. Techniques like single-cell RNA sequencing and pathomics offer deeper insights into the role of the TME. Liquid biopsy, as a minimally invasive method, enables real-time detection of circulating tumor components, facilitating the identification of predictive and prognostic biomarkers and illuminating tumor heterogeneity. These translational research advances are revolutionizing the understanding of cancer biology and are key to optimizing personalized treatment strategies. This review highlights emerging tools aimed at improving diagnostic and therapeutic precision in NSCLC, underscoring their role in decoding the interplay between tumor cells, the TME, and the host to ultimately improve patient outcomes.

## Simple Summary

Although therapeutic advances have improved outcomes for patients with non-small cell lung cancer, treatment resistance remains a major clinical challenge. To address this unmet need, innovative diagnostic and research strategies are being developed to better understand tumor biology and guide more effective, individualized treatment decisions. Techniques such as artificial intelligence, liquid biopsy, and single-cell analysis enable more precise characterization of tumors, offering promising avenues for the early detection of resistance, identification of predictive biomarkers, and advancement of personalized patient care. This review highlights how these emerging techniques could contribute to a more personalized and dynamic approach to lung cancer management, with potential implications for both clinical practice and future research directions.

## 1. Introduction

Lung cancer stands as the leading cause of cancer-related mortality worldwide and ranks second among the most diagnosed cancers in both men and women, according to the World Health Organization (WHO) cancer statistics [1]. Notably, data collected from the National Center for Health Statistics in the United States of America have emphasized that lung cancer had a higher mortality rate through 2021 than breast, prostate, and colorectal cancer combined [2]. This is largely the result of diagnosis at an advanced stage, leading to an unfavorable prognosis, with a 5-year survival rate of approximately 17% [3]. In addition, even though 16% of patients are diagnosed at an early stage, the vast majority experience disease relapse, progressing into the metastatic setting [4]. Despite its poor prognosis, there has been a significant shift over the past few years in the management of non-small cell lung cancer (NSCLC), particularly in screening, diagnosis, and treatment. The advent of molecularly targeted therapies, immune checkpoint blockade (ICB), anti-angiogenic agents, and, more recently, bispecific antibodies and antibody–drug conjugates (ADCs), has offered new hope in this challenging disease [5].

The development of ICB has transformed the treatment landscape across multiple cancer types. The long-term efficacy of ICB, which targets Programmed Death-1 (PD-1) and its ligand PD-L1, has been confirmed in various randomized phase III clinical trials involving patients with melanoma, NSCLC, and renal cell carcinoma (RCC), among others [6,7,8]. However, objective response rates with ICB monotherapy range between 20–40% across different tumor subtypes, indicating that while these treatments have improved overall cancer survival, a significant proportion of patients do not benefit from this strategy [9]. Furthermore, despite extensive efforts to identify predictive biomarkers of response to ICB, few have been validated, and their utility does not extend uniformly across all tumor types. The most commonly cited biomarkers are PD-L1 expression and microsatellite instability (MSI). For example, PD-L1 expression in tumor cells is associated with improved response and survival in patients with advanced NSCLC treated with pembrolizumab, but this correlation does not hold true for patients with melanoma or RCC [10]. On the other hand, MSI-high (MSI-H)—or mismatch repair deficiency (dMMR)—has become the first tumor-agnostic biomarker approved for ICB therapy, due to its predictive role across multiple tumor types [11]. However, the prevalence of MSI-H tumors remains low, limiting its benefit to a small subset of patients [12,13]. Other potential biomarkers, such as tumor mutational burden (TMB) or tumor-infiltrating lymphocytes (TILs), remain at an investigational level [14,15]. Despite these advances, reliable predictive biomarkers of response to immunotherapy remain an unmet need, prompting ongoing research into novel approaches to better assess and predict patient response.

Current biomarker discovery strategies often focus on the analysis of tumor-specific molecular pathways and rarely address tumor biology as a whole, primarily due to the inherent complexity of cancer biology. Artificial intelligence (AI) and liquid biopsy are progressively gaining ground in diagnosis, prognosis, and therapeutics, offering promising new avenues. Machine learning (ML) programs—a subset of AI—can learn from data to identify patterns and generate predictive models, thus providing a comprehensive analysis of histopathological samples through imaging features to assist in diagnosis and prognosis [16,17]. Other emerging techniques, such as the “omics” approaches—including pathomics and radiomics—are evolving fields that might contribute to a better understanding of tumor cells, their microenvironment, and their intricate relationship with the immune system and other host factors. Liquid biopsy, particularly the analysis of circulating tumor DNA (ctDNA), is emerging as a promising diagnostic, prognostic, and predictive biomarker, offering a non-invasive method of disease evaluation [18]. In this review, we present translational research methods and emerging techniques that contribute to a comprehensive understanding of immunotherapy within the field of oncology, with a particular focus on NSCLC.

## 2. The Host, Tumor Heterogeneity, and Tumor Microenvironment

The introduction of ICB into the therapeutic arsenal for NSCLC has significantly transformed the treatment landscape. However, durable clinical benefit is observed in only a subset of patients, underscoring the potential influence of host-related factors in modulating treatment efficacy.

The microbiota has been investigated as a potential biomarker of both tumorigenesis and treatment response. Current research in oncology has primarily focused on the role of intestinal microorganisms, which influence not only the efficacy of ICB treatment but also the development of treatment-related adverse effects [19,20]. In contrast, there is limited evidence regarding the impact of the lung or skin microbiome [21,22,23,24]. Nonetheless, studies in lung cancer patients and murine models suggest that dysregulation of the local lung microbiome may influence cancer progression by reshaping the tumor microenvironment (TME) and modulating the activity of infiltrating immune cells [25]. Strategies aimed at modulating the microbiota, such as the use of probiotics, prebiotics, or fecal microbiota transplantation, arise as promising approaches to enhance treatment outcomes [26].

In parallel, several host-related biomarkers have gained attention in the last decade as surrogates of prognosis in patients receiving immunotherapy. Parameters such as the neutrophil-to-lymphocyte ratio (NLR), the derived neutrophil-to-lymphocyte ratio (dNLR) or the Lung Immune Prognostic Index score (LIPI score) have demonstrated demonstrated value in capturing systemic inflammation and predicting therapeutic response and survival across multiple tumor types [27,28]. Moreover, understanding the molecular and genetic basis of intertumoral and intratumoral heterogeneity is crucial to elucidate the complex interplay among tumor cells, the immune system, the microbiota, and the host. While intertumoral heterogeneity reflects differences in histologically similar tumors among different patients, intratumoral heterogeneity describes molecular and phenotypic diversity within a single tumor specimen [29,30]. In fact, only 33% of somatic mutations are shared across all regions of the same tumor [31]. Non-genetic heterogeneity may arise from external and internal stressors, driving the emergence of novel subclones shaped by selective pressures in the microenvironment and interactions with immune, stromal, or extracellular matrix components [32,33].

Importantly, the inhibition of tumor-specific T cells by stromal myeloid and lymphoid cells, as well as the activation of inflammatory pathways, underscores the need to better characterize the complex crosstalk between tumor cells and a potential immunosuppressive TME in tumor progression and treatment resistance [34]. In this context, the analysis of single tissues and single cells has emerged as an interesting approach to dissect the cellular and molecular complexity of the TME. New working techniques, such as Single-cell RNA sequencing (scRNA-seq) and spatial RNA sequencing (spRNA-seq), together with translational techniques, including liquid biopsy or the use of AI, are increasingly used to resolve these challenges [35]. Furthermore, transcriptomic profiling of tumor-infiltrating immune cells enables the classification of immune subpopulations, identification of mechanisms of immune-escaping and drug resistance pathways, and supports the development of more effective targeted therapies, following or combined with immunotherapy [36]. These techniques, along with the identification of prognostic and predictive biomarkers of response to therapies, have revolutionized our understanding of cancer biology and might unravel new strategies for a more personalized approach.

## 3. Immune Checkpoint Blockers and Prognostic/Predictive Biomarkers

**Figure 1:**
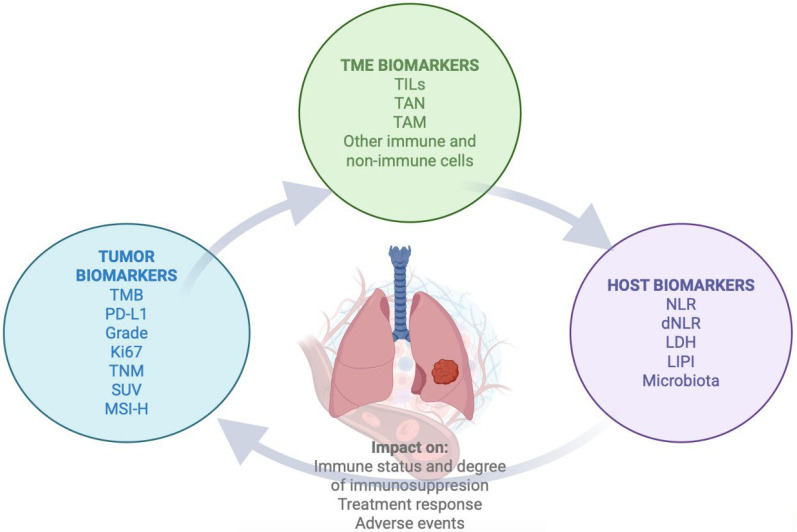
Prognostic and predictive biomarkers that have been previously described to impact ICB response in NSCLC. It is shown how the host, the tumor, and its microenvironment might also impact prognosis and response to ICB treatment, generating a complex interplay. Figure created with Biorender.com. NLR = Neutrophil/lymphocyte ratio; dNLR = derived NLR; LDH = Lactate dehydrogenase; LIPI = Lung immune prognostic score; TME = Tumor microenvironment; TILs = Tumor infiltrating lymphocytes; TANs = Tumor infiltrating neutrophils; TAM = Tumor associated macrophages; TMB = Tumor mutational burden; PD-L1 = Programmed-death ligand 1; TNM = Tumor, node, metastasis; SUV = Standardized Uptake Value; MSI-H = Microsatellite instability-high.

In recent years, ICB has transformed therapeutic strategies and significantly improved the prognosis of several solid tumors. In previously treated patients, ICB has demonstrated encouraging 5-year overall survival (OS) rates, reaching 34% in melanoma, 28% in RCC, and 16% in NSCLC, leading to approval of anti-PD-1, anti-PD-L1, and anti-Cytotoxic T Lymphocyte Antigen 4 inhibitor (anti-CTLA4) for its use in the metastatic setting across several malignancies [37]. Given these favorable outcomes, the use of ICB is now being extended to earlier disease stages, with the goal of enhancing curability and long-term survival [38]. Several biomarkers, such as TMB, PDL-1 expression, the microbiota, and the molecular and cellular characterization of the TME, have been associated with differential responses to immunotherapy, reinforcing their potential as predictive biomarkers [39,40,41]. Figure 1 summarizes key prognostic and predictive biomarkers currently under investigation or in clinical use for NSCLC [42].

### 3.1. PD1/PD-L1

PD-L1 was first described in the early 2000s in preclinical models, having been identified on antigen-presenting cells, endothelial cells, macrophages, and dendritic cells. It was subsequently recognized as the ligand of PD-1, a receptor primarily expressed on T cells, but also found on B cells and myeloid cells, where it exerts modulatory and inhibitory effects on immune function [43,44,45]. Initially considered as an interesting predictive biomarker of response to ICB, subsequent studies revealed limitations in its reliability, as some patients with low PD-L1 expression still derived clinical benefit in specific settings [46,47].

In NSCLC, the phase II Checkmate 063 was the first study to demonstrate the activity of anti-PD1 therapy with nivolumab in heavily pre-treated patients [48]. Since then, ICB has become a cornerstone in the management of metastatic NSCLC [49,50]. While PD-L1 expression was initially considered a strong predictor of response, subsequent evidence revealed that patients could respond regardless of PD-L1 status, challenging the biomarker’s utility as a sole determinant of treatment eligibility [46]. Later clinical trials confirmed the predictive value of PD-L1 expression in tumor cells, with the most pronounced efficacy observed in patients with expression levels ≥50%. Nevertheless, not all high expressors respond to therapy, and some patients with low/negative PD-L1 may still achieve meaningful clinical outcomes [51,52].

These findings highlight the complexity of PD-L1 as a biomarker and underscore the need for further research to elucidate the mechanisms underlying response variability and to identify more robust, reliable predictors of immunotherapy benefit.

### 3.2. Tumor Mutational Burden

TMB, defined as the number of somatic mutations per megabase (mut/Mb) of DNA, has been associated with improved treatment responses and survival outcomes in NSCLC patients receiving ICB therapy [53]. This association was notably supported by the CheckMate 227 study, the first phase 3 trial in lung cancer to evaluate immunotherapy efficacy in relation to TMB. In this trial, patients with high TMB (≥10 mut/Mb), who received first-line nivolumab and ipilimumab, demonstrated significantly longer progression-free survival (PFS), regardless of PD-L1 expression status [54].

Additionally, a retrospective analysis revealed that patients with both positive PD-L1 expression (defined as ≥1%) and high TMB had significantly better objective response rates (ORR) and PFS compared to those with only one or neither biomarker [55]. These findings suggest that TMB may provide additive predictive value to PD-L1 expression in identifying NSCLC patients most likely to benefit from immunotherapy, thereby enhancing the precision of treatment selection.

### 3.3. MSI-H

Among the few biomarkers that have demonstrated consistent predictive value across multiple tumor types, MSI-H stands out as the first to receive tumor-agnostic FDA approval for ICB. The underlying rationale stems from the observation that tumors with deficient DNA mismatch repair mechanisms accumulate a high mutational burden, leading to increased neoantigen production and enhanced immunogenicity—factors that improve response to PD-1/PD-L1 inhibitors. In the phase II KEYNOTE-158 basket trial, heavily pretreated patients with MSI-H/dMMR solid tumors achieved an ORR of 30.8% and a median duration of response of 47.5 months, findings that supported the tissue-agnostic approval of pembrolizumab [56].

Although MSI-H is rare in NSCLC—occurring in fewer than 1% of cases—it defines a distinct molecular subset of patients, often associated with a heavy smoking history, markedly elevated TMB, and recurrent loss-of-function mutations, most commonly in MLH1 [57]. Detection of MSI-H relies on immunohistochemistry (IHC) for mismatch repair proteins (MLH1, PMS2, MSH2, and MSH6), as well as Polymerase Chain Reaction (PCR)—or next-generation sequencing (NGS)-based microsatellite panels [58]. Due to its low prevalence in NSCLC, routine MSI testing of all cases remains controversial. However, targeted evaluation is recommended when broad-panel NGS reveals an unexpectedly high TMB or when IHC demonstrates unexplained loss of MMR protein expression.

### 3.4. Tumor Microenvironment and Host Biomarkers

Although the aforementioned biomarkers might contribute to improved clinical outcomes, a significant proportion of patients develop resistance to therapy or even fail to respond. Consequently, the identification of reliable predictive biomarkers remains an unmet need, particularly to discern which patients are most likely to benefit from PD-1/PD-L1 blockade. Focusing on the host and tumor microenvironment, TILs, tumor-associated macrophages (TAMs), tumor-associated neutrophils (TANs), blood-based inflammatory biomarkers—such as the platelet-to-lymphocyte ratio (PLR), NLR or dNLR, and LIPI score-, and the microbiota, arise as potential strategies for better selection of patients and outcomes predictions [59,60,61].

Notably, the emergence of several host and TME-related biomarkers in recent years reflects a conceptual shift: markers previously regarded as purely prognostic are now being explored for their predictive potential in immunotherapy. This shift underscores the growing recognition that components of the tumor-immune interface may actively modulate therapeutic response, rather than merely reflect disease burden or biology. TILs, traditionally viewed as prognostic, are now gaining recognition for their predictive value. Their density and spatial organization within the TME may indicate a pre-existing antitumor immune response and an inflamed TME, both of which have been associated with improved outcomes following ICB in multiple tumor types, including NSCLC [62,63,64]. Similarly, the presence of TAMs, TANs, and other immune and stromal components of the TME contributes to the immunological milieu and may influence immunotherapeutic efficacy. Previous studies have correlated elevated expression of TAMs, particularly the M2 phenotype, and TANs with poor prognosis [65,66,67]. Beyond their prognostic value, accumulating evidence supports their role in therapeutic resistance. M2-polarized TAMs promote resistance to chemotherapy, targeted therapies, radiotherapy, and immunotherapy in NSCLC by activating immunosuppressive pathways such as JAK/STAT, PI3K/AKT, and TGF-β/Smad [68]. Through cytokine secretion, exosomal signaling, and interactions with cancer stem cells and immune populations, TAMs may remodel the TME to favor tumor survival and immune evasion [69]. Combination strategies targeting TAM-related mechanisms thus show promise in overcoming therapeutic resistance [70]. In parallel, the predictive role of TINs is receiving increasing attention. Specific neutrophil phenotypes and gene expression profiles—such as CD74+high neutrophils—have been linked to enhanced antitumor immunity and may predict responsiveness to immunotherapy, either alone or in combination with chemotherapy [71,72]. These findings support the incorporation of TAM and TIN profiling into integrative biomarker models to refine risk stratification and guide treatment decisions in NSCLC.

The role of the microbiota as a biomarker in NSCLC is gaining increasing attention in the field of translational oncology. Emerging evidence suggests that the composition and diversity of the microbiome may influence tumor initiation, progression, and response to therapy [73,74]. Specific bacterial genera, such as *Haemophilus*, *Prevotella*, and *Streptococcus* have been found in greater abundance in lung cancer patients [75]. Such dysbiosis may contribute to carcinogenesis by promoting chronic inflammation, altering host immune responses, and activating oncogenic signaling cascades. Mechanistically, the microbiota can modulate DNA damage repair, mutagenesis, and cell cycle regulation [76]. Metabolomic studies have further linked alterations in microbial metabolites—such as those involved in sphingolipid and autophagy pathways—to lung cancer pathophysiology [77,78]. Furthermore, the microbiota may impact therapeutic outcomes, particularly in patients receiving ICB, by shaping the tumor immune microenvironment [74,79]. Despite these promising insights, the field remains in early stages, and larger, prospective studies are needed to validate microbial biomarkers. Ultimately, integrating microbiota profiling into clinical practice could contribute to more personalized approaches in lung cancer diagnosis, prognosis, and treatment.

### 3.5. Other Biomarkers

In addition to molecular and immune-related biomarkers, several host- and tumor-specific factors play a pivotal role in the prognosis and therapeutic stratification of NSCLC. Established clinicopathological parameters such as tumor grade, TNM stage, standardized uptake value (SUV) from FDG-PET imaging, and proliferative index markers like Ki-67 continue to provide critical prognostic and predictive information. High Ki-67 expression and elevated SUV are generally associated with more aggressive disease and poorer outcomes, while TNM staging remains a cornerstone for treatment decisions [80,81]. Integrating emerging translational biomarkers with established clinical and pathological indicators may significantly improve the precision of patient stratification and facilitate a more comprehensive and personalized approach to the management of NSCLC.

Nevertheless, despite growing evidence, the intricate interplay between tumor cells, the host immune system, and the TME presents significant challenges for the routine clinical implementation of these biomarkers. In this context, AI and advanced translational research methodologies offer valuable tools for deciphering complex biological data and identifying robust, clinically applicable predictive biomarkers.

## 4. New Pathology Techniques in the Translational Oncology Era

**Figure 2:**
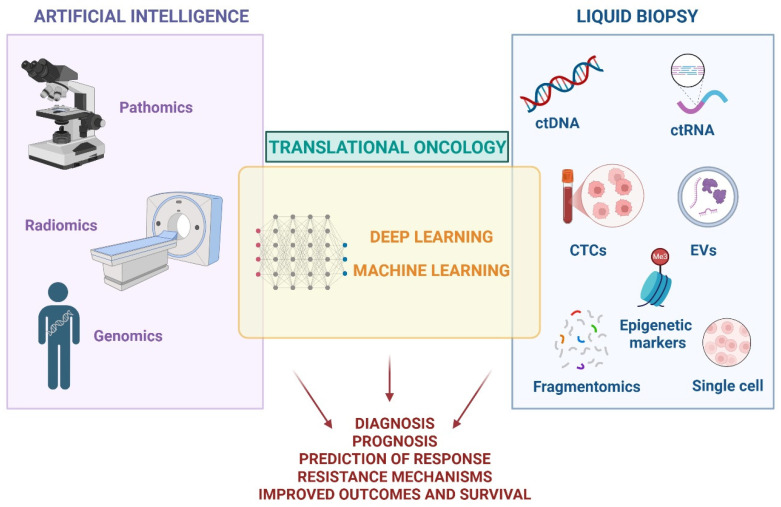
New pathology techniques in translational oncology era, which may influence patient approach and treatment strategies. Figure created with Biorender.com. ctDNA = circulating-tumor DNA; ctRNA = circulating-tumor RNA; CTCs = circulating tumor cells; EVs = extracellular vesicles.

AI and liquid biopsy are currently emerging as promising tools for advancing precision and personalization in cancer care (Figure 2). However, despite their potential, AI has not yet been broadly integrated into routine clinical workflows, and liquid biopsy—though implemented in some centers—still requires broader validation, standardization, and regulatory approval. Both technologies remain active areas of research and hold considerable promise for enhancing diagnostic accuracy, optimizing treatment decisions, and ultimately improving patient outcomes and quality of life.

### 4.1. Artificial Intelligence

AI is defined as the capability to predict or classify objects through algorithmic analysis of existing data. This transformative technology includes disciplines such as machine learning (ML) and deep learning (DL), which are transforming the management of health care and other industries by optimizing complex data analysis [82]. ML develops self-learning algorithms from its accumulated experience and solves complex problems without explicit programming to enhance its performance, improving in fields such as natural language processing, computer vision, and recommendation systems. DL is composed of multiple-layered ML models that achieve feature selection and model fitting simultaneously, requiring a large database input capable of dealing with more complex patterns [83,84]. Mimicking the structure and functionality of the human brain, DL has led to remarkable breakthroughs in complex tasks, such as image and speech recognition, language translation, and self-driving cars [85].

In oncology, numerous studies have reported the applicability of AI in diagnostics, therapeutics, and prognostic predictions in several neoplasms [86,87,88]. The so-called “omics” approach, namely pathomics, radiomics, and genomics, is increasingly evolving, representing a new pathway in translational techniques. Radiomics extracts quantitative information from radiological images, identifying complex patterns that may provide information on tumor heterogeneity and determine prognosis [89]. Pathomics focuses on the analysis of histopathological images to extract quantitative features and characterize tissue samples in whole slide images (WSI). As a result, it allows spatial characterization of tumor and stroma, shapes and texture of nuclei, characterization of lymphocytic infiltrates and other cell types, and identifies different biomarkers by IHC [90]. Genomics provides interpretation of gene expression patterns and dynamics, helping in the identification of prognostic biomarkers, as well as molecular mechanisms of resistance [91]. These multi-omics approaches, when combined with ML and DL, enhance the understanding of cancer biology and refine diagnostic and prognostic capabilities.

Importantly, patient-derived xenograft (PDX) models have emerged as valuable tools for validating insights gained from multi-omics. As reported by Gu et al. (2025) [92], PDX models retain the genetic and phenotypic complexity of the original human tumors, providing an in vivo platform for integrated molecular profiling and functional validation. When coupled with high-throughput sequencing, PDXs allow researchers to explore tumor heterogeneity at single-cell resolution and to identify rare subclones and resistance pathways that may be missed in bulk analyses. These models serve as a bridge between computational predictions and biological relevance, reinforcing the clinical utility of AI-driven omics research in oncology [92].

Indeed, both ML and DL have the potential to impact every stage of oncology care—from diagnosis and prognosis to treatment selection and disease monitoring [93]. However, their clinical implementation faces significant hurdles, including variability in regulatory standards, inconsistent model evaluation practices, limited interpretability, and challenges in real-world integration. Discrepancies between regulatory frameworks—such as those of the United States Food and Drug Administration (FDA) and the European CE mark system—raise concerns about premature clinical deployment and under-regulation of ML-based tools [93]. Moreover, the lack of large, diverse, and well-annotated validation datasets limits model generalizability and may introduce bias when applied across different patient populations [94]. Despite these challenges, as AI systems become more transparent, clinically validated, and supported by harmonized oversight, their potential to reshape precision oncology continues to grow.

In NSCLC, AI and multi-omics approaches have shown promise in improving prognostic accuracy. Several studies have demonstrated improved prediction of 1-year PFS among patients treated with immunotherapy using AI-enhanced models [95,96]. Furthermore, these tools have demonstrated diagnostic utility by increasing sensitivity and specificity in the detection and characterization of pulmonary nodules on conventional imaging, thereby enabling earlier and more accurate diagnosis. AI models have also shown potential in predicting *Epidermal Growth Factor Receptor* (EGFR) mutation status and estimating TMB, both of which are critical for treatment planning and patient stratification [97].

To summarize, the integration of AI technologies—including ML, DL, and multi-omics frameworks—into cancer research and clinical practice holds immense promise for improving diagnostic precision, accelerating therapeutic discovery, and advancing personalized medicine [98].

#### Artificial Intelligence in Pathology Diagnosis

AI techniques applied to digital pathology involve converting conventional glass slides into WSI, enabling centralized storage, remote access, and advanced computational analysis [99]. Slides generated by DICOM WSI can encompass more than 4 billion pixels at 40× magnification, scanning at a resolution of 0.25 μm/pixel. This extraordinary level of detail provides a detailed visualization of cellular and tissue structures [100].

The applicability of WSI in NSCLC can effectively differentiate between histological subtypes and identify molecular alterations like *STK11, EGFR, FAT1, SETBP1, KRAS,* and *TP53* mutations. A study by Coudray et al. (2018) [101] showed an impressive accuracy in predicting molecular phenotype, ranging from 73% to 85.6% for *EGFR* and *KRAS* mutations, two of the most important targetable pathways in NSCLC, making a profound impact on personalized treatment strategies. Another study published by Yu et al. (2016) [102], which gathered 2186 full-scan images from patients with lung adenocarcinoma and squamous cell carcinoma, employed ML on histopathology WSI. They successfully characterized and identified morphologic features that assisted pathologists in distinguishing malignant tumors from healthy tissue, as well as predicting survival outcomes. Features that correlated with survival in this ML model included the shape of the nuclei and cytoplasm [102].

A recent study further advanced the field by training convolutional neural networks to classify lung adenocarcinoma subtypes and predict disease-specific survival. Using consensus images from 17 expert pathologists, the DL models demonstrated high accuracy across eight lung adenocarcinoma subtypes and were independently validated in a cohort of 133 patients [103]. At the same time, advances in scalability and efficiency have led to the development of DL models that can be trained on entire WSIs using only slide-level labels, eliminating the need for labor-intensive, detailed annotations. In a dataset of 9662 lung cancer WSIs, this approach achieved impressive area under the curve (AUC) values of 0.9594 for adenocarcinoma and 0.9414 for squamous cell carcinoma, outperforming traditional multiple-instance learning methods and successfully localizing small lesions through class activation mapping [104].

In a comprehensive systematic review, Davri et al. (2023) [105] examined 96 studies applying DL to histologic and cytologic images in lung cancer, concluding that these tools significantly improve diagnostic workflows, enhance the reproducibility of subtype classification, and support predictive and prognostic decision-making based on tissue morphology.

In the context of biomarker identification, PD-L1 expression in NSCLC has also been assessed using computer-assisted scoring based on hematoxylin-eosin WSI. This model managed to accurately predict PD-L1 status by correlating PD-L1 expression with morphological features in TME [106].

This accumulating body of evidence underscores how AI can serve as a powerful ally to pathologists, aiding in the diagnosis, phenotyping, and prognostic prediction of lung cancer by analyzing digital pathological sections. With AI assistance, diagnostic efficiency may be increased exponentially, reducing inter-observer variations and inaccuracies intrinsic to human visual interpretation. As AI continues to evolve, its transformative impact on healthcare holds the promise of providing a more accurate overview of lung cancer management. The ML and DL models applied to WSI and histopathology aim to enhance the accuracy not only of diagnostic procedures, but also of risk prediction, response detection, and treatment outcomes assessment. However, the standardization and routine clinical integration of AI techniques still require further research to validate their reliability, reproducibility, and practical applicability.

### 4.2. Liquid Biopsy

Liquid biopsy refers to the detection and analysis of tumor-derived components present in body fluids, offering a minimally invasive alternative to conventional tissue biopsy [18,107]. Its non-invasive nature allows for repeated sampling at multiple time points, enabling timely and cost-effective monitoring of treatment response, detection of resistance mechanisms, and assessment of disease relapse or progression in NSCLC patients [108]. In addition, liquid biopsy enables the identification of new treatment targets and biomarkers, shedding light on promising avenues for the development of novel therapies [109]. As this technology continues to evolve, its integration into routine clinical practice holds the promise of greater accuracy and improved patient outcomes, moving towards a more personalized approach.

#### 4.2.1. Sample Types

Liquid biopsy involves the analysis of circulating tumor cells (CTCs) and tumor-derived molecules, including ctDNA, circulating-tumor RNA (ctRNA), microRNA (miRNA), or extracellular vesicles (EVs). By capturing critical genetic, epigenetic, and proteomic changes, it represents an opportunity to identify tumor-specific alterations and genetic mutations that drive cancer growth and therapy resistance. Although the blood is the most common source for liquid biopsy, other body fluids can be used, such as urine, sputum, saliva, and cerebrospinal fluid [110].

##### Circulating Tumor Cells

CTCs are isolated tumor cells within the bloodstream or body fluids, though often present in very low concentrations. Various methods have emerged to improve their detectability. The Isolation by Epithelial Tumor Cell Size (ISET) approach emerged as one of the first size-based methods for CTC detection. This technique provides morphological, immunocytological, and genetic characterization of CTCs, with exceptional sensitivity and repeatability [111]. In parallel, techniques like flow cytometry, fluorescence-activated cell sorting (FACS), and microfluidics have gained prominence, providing powerful tools to efficiently isolate CTCs from liquid biopsy samples [112,113]. Among these methods, the widely used epithelial cell adhesion molecule (EpCAM) immunomagnetic assay allows for the enumeration of CTCs of epithelial origin in the blood. However, it may miss a substantial cell population with “stem cell-like” properties, warranting further exploration into alternative approaches. An emerging strategy known as laser capture microdissection involves encapsulating a CTC in a hydrogel, enabling targeted extraction using a laser. This is followed by sequencing, thus providing its genetic and molecular profile [114]. Continued research into innovative techniques for CTC detection and characterization holds the potential to enhance our understanding of cancer biology.

Focusing on the applicability of CTCs in lung cancer, their detection in patients diagnosed with NSCLC is frequently associated with poor prognosis, emphasizing the critical role they play in disease progression and treatment response [18]. Although they are detectable in only 30% of patients, they have been associated with worse response and survival in metastatic patients treated with ICB [115]. Several prospective studies have also assessed the prognostic role of CTCs in first-line chemotherapy-treated patients, demonstrating worse OS and PFS in the presence of a higher load of cancer cells [116,117]. In the early setting, detectability of CTCs pre- and post-operatively has been correlated to shorter disease-free survival [118,119]. Regarding their diagnostic utility, a meta-analysis of 21 studies involving 2714 patients reported that CTCs have a sensitivity of 72% and specificity of 96% for the diagnosis of NSCLC, supporting their clinical relevance [120].

Overall, the available evidence underscores the potential of CTCs as a valuable diagnostic and prognostic tool in the management of lung cancer, offering a non-invasive approach for early detection and monitoring of the disease.

##### Circulating Tumor DNA

CtDNA is a component of the circulating-free DNA originating from tumor cells that have undergone apoptosis or necrosis. It has been previously described as a prognostic and predictive biomarker in various tumor types and settings, including neoadjuvant, adjuvant, and metastatic disease [121,122,123]. Given the correlation between ctDNA levels and tumor burden, it represents an invaluable biomarker for disease monitoring and assessment of prognosis [124,125]. Classically, the quantification and analysis of ctDNA is carried out by PCR-based or NGS-based techniques [126]. These methods represent a non-invasive and easily accessible means of capturing tumor-derived genetic material.

Current evidence highlights its use in screening, early diagnosis, detection of minimal residual (MRD) disease after radical treatment, assessment of the risk of metastasis and recurrence, and identification of resistance mechanisms in NSCLC [127]. In a retrospective study from the Impower131 cohort including 221 patients diagnosed with metastatic NSCLC treated with chemo-immunotherapy, the ctDNA decrease from baseline was associated with improved outcomes, including better PFS and OS. Conversely, any increase in ctDNA levels during serial monitoring correlated with worse PFS and OS [128]. Similarly, an international phase II trial of patients treated with first-line pembrolizumab demonstrated a strong concordance between ctDNA dynamics and radiological response. The study reported a sensitivity of 82% and a specificity of 75% when comparing ctDNA clearance with objective response based on RECIST criteria. Notably, patients who achieved ctDNA clearance experienced significantly longer PFS and OS compared to those with persistently detectable ctDNA [129].

Notably, ctDNA has also demonstrated the ability to detect molecular relapse before clinical or radiological signs, allowing for earlier intervention and reduced exposure to ineffective treatments [130]. Unlike tissue biopsy, which captures information from a single site, ctDNA reflects heterogeneity across multiple tumor clones, offering a more comprehensive overview of tumor evolution under therapeutic pressure [110]. An example of this was described by Rolfo et al. (2021) [131], who reported that ctDNA analysis enabled the detection of resistance mutations—such as *EGFR T790M*—in NSCLC patients, facilitating timely treatment modifications without requiring repeat biopsies.

The BFAST trial further highlighted the role of ctDNA in the upfront detection of actionable mutations. In this study, ctDNA successfully identified *Anaplastic Lymphoma Kinase* (ALK) rearrangements in treatment-naive NSCLC patients, enabling the initiation of targeted therapy and achieving response rates comparable to tissue-based testing [132]. Additional studies have supported the predictive role of ctDNA in oncogene-driven NSCLC. For example, the presence of the *T790M* mutation at progression in *EGFR*-mutant patients treated with osimertinib, an EGFR- tyrosine kinase inhibitor (TKI) inhibitor, was associated with worse outcomes [133]. In *ALK*-rearranged NSCLC, a phase II trial demonstrated that detection of ALK translocation in plasma or tissue after TKI failure predicted better responses to lorlatinib, an ALK-TKI inhibitor, while its absence suggested off-target resistance mechanisms [134].

In earlier-stage disease, preoperative ctDNA positivity has also been linked to poorer outcomes. A systematic review and meta-analysis by Lu et al. (2024) [135] found that ctDNA detection in patients with stage I–II lung adenocarcinoma correlated with shorter recurrence-free survival and OS, while this association was not significant in stage III disease. Moreover, ctDNA monitoring post-surgery for MRD may offer high specificity in predicting relapse, though its sensitivity remains suboptimal [136].

Despite promising evidence, ctDNA implementation in clinical practice is not yet fully standardized. Expert guidelines underscore its utility for MRD detection and genomic profiling, particularly when tissue biopsy is infeasible or insufficient [137,138,139]. However, both ctDNA and tissue-based analyses carry non-negligible false-negative rates, reinforcing the need for complementary testing strategies to improve turnaround time and detection of targetable alterations [139].

Overall, as the understanding of ctDNA continues to deepen, its applicability becomes increasingly evident in various clinical settings due to its prognostic and predictive value. The integration of ctDNA-based approaches into routine clinical practice holds the promise of improving patient outcomes through personalized treatment strategies. However, there is insufficient robust data yet for its widespread implementation and validation across diverse patient populations.

##### Circulating Tumor RNA

CtRNA, although less commonly used than ctDNA in liquid biopsy, has been described as an interesting diagnostic and prognostic biomarker. Its detection in the bloodstream, mainly in the form of long non-coding RNA (lncRNA), messenger RNA (mRNA), or miRNA, provides information on molecular changes linked to cancer development and progression, unveiling pathways that may reveal novel therapeutic strategies [140]. CtRNA analysis can be performed by digital-droplet PCR (ddPCR), quantitative reverse transcription PCR (RT-qPCR), microarrays, and NGS methods such as RNA sequencing (RNAseq) or small RNAseq. NGS-based methods offer several advantages, including a broad dynamic range, minimal sample requirements, high reproducibility, and the ability to detect novel transcripts even in the absence of a reference genome [141]. This has facilitated the identification of diagnostic, prognostic, and predictive biomarkers—such as lncRNAs, miRNAs, and circular RNAs (circRNAs)—as well as markers of drug resistance and neoantigen profiling [142].

Evidence focusing on miRNAs in NSCLC supports their role as diagnostic tools in distinguishing histological subtypes. For example, increased levels of miR-181-5p and miR-361-5p have been observed in adenocarcinoma compared to squamous cell carcinoma. Additionally, some miRNAs have been associated with poor prognosis and treatment resistance. Low levels of miR-146-5p have been correlated with cisplatin resistance and worse PFS in NSCLC [143]. A study by Liu et al. (2016) [144], conducted on 10 patients diagnosed with NSCLC, evaluated the expression of miRNAs using a quantitative PCR array panel and found that several exosomal miRNAs, including miR-23b-3p, miR-10b-5p, and miR-21-5p, were significantly associated with poor OS. Regarding circRNA -a type of non-coding RNA- it has been previously associated with cancer development and progression. A study identified 357 dysregulated circRNAs in lung cancer patients. Overexpression of certain circRNAs was linked to treatment resistance, such as the presence of cic-CCDC66 in EGFR-inhibitor resistant patients or circ-ABCB10 in Cisplatin-resistant lung cancer [145].

Beyond their utility as biomarkers in liquid biopsy, RNAs are also being explored as active therapeutic agents in cancer immunotherapy. Recent advances in mRNA engineering and delivery technologies have enabled the development of mRNA-based vaccines and therapeutics that can stimulate potent anti-tumor immune responses [146]. These strategies include the delivery of tumor-specific antigens, cytokines, or immune-modulating factors via synthetic mRNA platforms. Optimized structural design and nanoparticle-based delivery systems have further improved the stability, translational efficiency, and targeting of therapeutic mRNAs [147]. This dual role of RNA—both as a biomarker and as a therapeutic vector—illustrates its growing relevance in precision oncology and highlights the potential for ctRNA analysis not only in cancer detection but also in guiding and monitoring RNA-based immunotherapies.

Overall, ctRNA represents an interesting field of research to enhance clinical outcomes by the detection of diagnostic, prognostic, and predictive biomarkers, as well as treatment resistance factors.

##### Extracellular Vesicles

Extracellular vesicles (EVs) are small, membrane-bound products derived from the cell’s endosomal system, carrying DNA fragments, non-coding RNAs, mRNAs, and proteins. Methods for their detection include conventional protein analysis techniques, such as Western blot and ELISA, and nucleic acid analysis techniques, including RT-qPCR, ddPCR, and NGS [148]. Previous research has highlighted their significance as diagnostic and prognostic biomarkers, in addition to their role as mediators of tumorigenesis and metastasis [149].

A study involving 179 patients diagnosed with NSCLC observed an upregulation of serum-derived exosomal LINC00917 in stage III-IV, which correlateded with poor OS [150]. Another study, which collected blood samples from 66 lung cancer patients, identified an increase in PLA2G10, a protein incorporated in EV, in tumors exhibiting more aggressive features such as higher stage or presence of distant metastasis. An association between high levels of PLA2G10 and worse OS and recurrence-free survival was also found [151]. Moreover, several EVs have been previously described as diagnostic biomarkers, such as CDL5-, or therapeutic targets, such as ITIH4, SERFINF1, SAA4, SERFINC1, and C20ORF3. This represents an interesting and non-invasive cancer approach for cancer detection and personalized cancer treatment [152].

To sum up, the detection of EVs is a promising strategy to further enhance patient outcomes. Together with ctDNA, ctRNA, and CTCs, these approaches offer valuable insights into the dynamic changes of cancer, and provide non-invasive methods for monitoring cancer progression, treatment response and prognosis.

#### 4.2.2. Liquid Biopsy Techniques

While several analytical platforms are available for liquid biopsy, the most commonly employed techniques are PCR-based methods and NGS, particularly for ctDNA assessment. Due to their reliability, scalability, and increasing accessibility, both methods have become the cornerstone of ctDNA analysis in clinical and research settings [107].

##### PCR-Based Techniques

PCR-based techniques are one of the most frequent techniques used in liquid biopsy, capable of detecting hot-spot mutations with driver properties and therapeutic implications. Nevertheless, it requires the presence of already-known specific mutations, restricting the ability to uncover novel genetic alterations or resistance mechanisms that may hold critical clinical significance [153].

PCR-based approaches include digital PCR (dPCR) platforms, such as real-time PCR (RT-PCR) and BEAMing—acronym for Beams, Emulsions, Amplifications, Magnetics-, and ddPCR. RT-PCR is a semiquantitative method for analysis with an affordable price and lower sensitivity compared to ddPCR techniques, but it has high specificity for detecting known point mutations. Sensitivity varies greatly depending on the technique and type of tumor, which makes it challenging when applied to clinical practice [154]. On the other hand, ddPCR techniques are highly sensitive and more complex methods allowing the detection of rare events and the quantification of a single molecule. However, they have a higher risk of contamination due to multiple transfer and pipetting steps, requiring the presence of specialized scientists for their proper use [155].

Emerging techniques adapted from PCR-conventional methods based on multiplex detection include mass-spectrometry, which is ultrasensitive for detecting low-frequency mutations with as low as 0.1% minor allele frequency. UltraSEEK (high-throughput, multiplexed, ultrasensitive mutation detection) and SERS (Surface-Enhanced Raman Spectroscopy) are two mass-spectrometry techniques that require a low input of ctDNA for analysis and achieve great sensitivity and specificity targeting 1–3 mutations [156,157,158].

##### NGS-Based Techniques

NGS-based-techniques are a powerful tool to identify multiple mutations across large genomic regions, achieving remarkable sensitivity and specificity. Indeed, techniques as Massively Parallel Shotgun Sequencing and Massively Parallel Sequencing offer the ability to quantify copy number alterations, informing on tumor genomic complexity and heterogeneity. However, its accuracy depends on the platform used, as there are various methods and profiles, including deep-sequencing, multiplex-PCR NGS, Cancer Personalized Profiling by deep-sequencing (CAPP-Seq), Tagged-Amplicon deep sequencing (TAm-seq), Safe-Sequencing System (Safe-SeqS), among others. These techniques require experienced bioinformaticians to analyze and interpret the data obtained, which is both expensive and time-consuming [158,159].

Two primary types of sequencing panels are commonly available for liquid biopsy analysis: targeted and untargeted panels. Targeted panels are meticulously designed to detect specific genomic sequences, offering high sensitivity in capturing low-frequency mutations, while they may miss mutations that lie beyond the pre-defined regions, thus presenting limitations in detecting new or unexpected mutations. In contrast, untargeted panels, also known as whole-genome sequencing (WGS) or whole-exome sequencing (WES), encompass a genome-wide or exome-wide exploration, offering the potential to discover previously unknown or unforeseen mutations that may offer insights into patient prognosis or new treatment strategies. However, untargeted panels may have limited sensitivity in detecting low-frequency mutations, which remains a consideration in their clinical implementation [160,161].

#### 4.2.3. Liquid Biopsy Workflow

Each liquid-biopsy modality has distinct technical requirements: ctDNA workflows hinge on rapid plasma separation and rigorous nucleic-acid stabilisation; ctRNA and circRNA workflows demand stringent RNase inhibition and tight temperature control to preserve the more labile RNA species; while CTC protocols focus on maintaining cellular integrity through specialized preservative media followed by physical or immuno-enrichment [162].

Several pre-analytical and analytical variables critically influence the accuracy and reliability of liquid biopsy results and should therefore be standardized in routine clinical practice, as outlined by the European Society for Medical Oncology (ESMO) guidelines [163]. The timing of blood sampling must align with the clinical objective. For the assessment of MRD after surgery, blood should be collected at least 7 days postoperatively—or ideally, 14 days after major resections—to avoid contamination by cell-free DNA (cfDNA) released during wound healing. For genotyping in advanced disease, sampling should be performed outside of periods of active, radiologically confirmed treatment response, as effective therapies may suppress ctDNA shedding, increasing the likelihood of false-negative results. Regarding sample handling, EDTA tubes are appropriate when plasma can be processed within four hours. If immediate processing is not possible or if samples require transport to external laboratories, cfDNA-stabilizing tubes—permitting delays of up to seven days—are preferred. For long-term storage, plasma should be maintained at −80 °C with minimal temperature fluctuations, and repeated freeze–thaw cycles should be avoided to preserve nucleic acid integrity [163].

Despite rigorous sampling and handling protocols, false-negative results can still occur in liquid biopsy analyses. These may arise from low ctDNA abundance, limited assay sensitivity, or the presence of intrinsically “non-shedding” tumors that release minimal detectable DNA into circulation [164]. Conversely, false positives are frequently attributed to clonal hematopoiesis of indeterminate potential (CHIP), especially when mutations are detected in DNA repair or tumor suppressor genes such as *TP53* or *DNMT3A* [165]. To mitigate this, routine collection and parallel sequencing of the buffy coat—enriched in leukocytes—are recommended to distinguish CHIP-related variants from true tumor-derived mutations. Pathogenic germline alterations in genes such as *BRCA1*, *BRCA2,* or *PALB2* may also surface in plasma, necessitating reflex germline confirmation with a validated assay. Finally, clinical ctDNA workflows should incorporate computational algorithms or orthogonal purity assessments to estimate tumor-derived DNA content. This would enhance confidence in negative results and guide decisions on the necessity of complementary tissue biopsy [163].

Meticulous control of these variables—coupled with rigorous external-quality assessment—will maximize the sensitivity and specificity of ctDNA testing, minimize interpretative pitfalls, and facilitate its seamless integration into precision-oncology workflows for NSCLC.

#### 4.2.4. Emerging Methods for Liquid Biopsy Biomarker Discovery

##### Long-Read Sequencing

New sequencing methods involve third-generation technologies with longer sequencing reads, shorter preparation time, and the possibility of sequencing RNA and detect nucleic acid modifications. In addition, they allow the detection of single-nucleotide variants (SNVs) and small insertion-deletion mutations (indels) in liquid biopsies, surpassing short-read techniques. However, an error rate of 10–15% can be expected when using long-read sequencing, which can be reduced by multiple sequencing runs [166,167].

##### DNA Methylation Markers

DNA methylation is one of the hallmarks of cancer, and it is also the first and most studied epigenetic mark in cancer. Methylation signatures observed in tumor tissue are known to occur early in carcinogenesis and promote tumor progression. As a result, it has been proposed as a potential screening and diagnostic tool to improve early cancer detection [168]. A study carried out by the Circulating Cell-free Genome Atlas, which involved 2800 participants, compared different cfDNA approaches using ML to evaluate their precision and limit of detection. Remarkably, genome-wide methylation analysis outperformed WGS and targeted sequencing approaches, emerging as a promising genomic feature for cancer signal detection. This technique managed to accurately predict cancer signal origin with greater accuracy than other techniques [169].

As our understanding of epigenetic alterations expands, methylation technologies appear as transformative tools in precision oncology with the potential of assessing early detection and diagnosis of cancer. However, several challenges arise. Methylation occurs not only in cancer cells but also in normal cells, increasing its frequency with age. Additionally, differences among tumors and certain scenarios, such as myocardial infarction, can also promote epigenetic alterations, potentially leading to difficulties when applying these techniques [170].

Overall, although emerging techniques based on methylation markers unravel epigenetic changes associated with tumor development and progression and can thus increase sensitivity, they still require further research for validation to enhance their applicability in clinical practice [171].

##### Single-Cell Sequencing

Single-cell analysis has emerged as an interesting tool for CTCs and cancer-associated immune cells analyses. These sequencing techniques include scRNA-seq, which provides insight of the gene expression signatures of individual cells, and single-cell DNA sequencing (scDNA-seq), which helps identify single-nucleotide variants, copy number variations, or MSI. Other technologies, such as single cell proteomics, epigenomics, or metabolomics, provide information on the metabolic pathways within tumor cells, but still require further research for their widespread adoption [172,173].

While scDNA-seq allows the recognition of mutations throughout the genome of a single cell, it has limitations in detecting significant expression differences in heterogeneous cells within the TME. In contrast, scRNA-seq not only reveals intercellular gene expression differences but also identifies heterogeneity within phenotypically distinct immune and stromal cell populations. In NSCLC, scRNA-seq has uncovered a high degree of T cell diversity, including multiple CD8+ and CD4+ T cell subsets [174]. A key finding from these studies is the role of B cells in establishing tertiary lymphoid structures (TLS) within the TME—facilitating lymphocyte maturation, immune activation, and clonal expansion of both T and B cells [175]. Furthermore, scRNA-seq has shed light on the plasticity of TAMs, dendritic cell heterogeneity, and neutrophil remodeling within the tumor milieu [176,177].

Recent advances in single-cell analysis have significantly enhanced our understanding of the immune microenvironment in NSCLC. By allowing high-resolution mapping of intratumoral cellular components, these techniques provide a robust framework for identifying immune subpopulations that influence therapeutic response and resistance. This detailed profiling supports the design of more personalized immunotherapeutic strategies [178].

Leader et al. (2021) [179] contributed to this field by identifying the “lung cancer activation module” (LCAM), an immune signature composed of activated T lymphocytes, IgG plasma cells, and SPP1+ macrophages. The presence of LCAM is associated with a higher likelihood of response to immunotherapy, even among patients with high TMB, suggesting that immune cell composition provides non-redundant predictive information beyond genomic biomarkers. Moreover, multimodal analysis and spatial data integration have enabled the discovery of lymphocyte phenotypes and patterns of cellular organization that are predictive of response to ICB, thereby improving therapeutic selection in NSCLC [180,181].

Notably, single-cell technologies have also elucidated mechanisms of treatment resistance. In *EGFR*-mutant NSCLC, single-cell analyses have identified clonal evolution and transcriptomic changes linked to resistance against tyrosine kinase inhibitors. These studies have highlighted immune remodeling and transcriptional reprogramming as central features of acquired resistance, paving the way for more precise and adaptive treatment strategies [182,183].

Overall, single-cell analysis techniques, and particularly scRNA-seq, enable the characterization of tumor heterogeneity, thus providing insights into potential biomarkers of clinical application. However, they require meticulous protocols and quality control measures. Library construction, after cell isolation, needs whole genome or whole transcriptome amplification (WGA/WTA) to generate sufficient genetic material for sequencing [167]. As a result, they require further investigations to fully understand their potential in cancer research and their clinical applications.

##### Fragmentomics

Fragmentomics in liquid biopsy is a highly promising and innovative approach for cancer detection and monitoring. This new field focuses on the study of fragments of circulating DNA (circDNA), which are the result of genetic material released during necrosis, apoptosis, or phagocytosis [184]. This fragmentation pattern correlates with nucleosome positioning and gene expression, thus providing valuable information on genetic and epigenetic features [185]. Previous evidence highlighted the role of circDNA to discriminate between healthy individuals and cancer patients, and to identify specific mutations depending on the length of fragments. In fact, cancer patients have shorter median lengths and greater variability in the size of these fragments [186]. Mouliere et al. (2018) [187] observed that the identification of size-selected circDNA allowed the detection of actionable mutations and copy number alterations that had not been identified by other methods.

Fragmentomics has been evaluated for early diagnosis, surveillance, detection of residual disease, and identification of resistance mechanisms in NSCLC. A study of 350 healthy individuals and 432 patients with cancer merged circDNA features with five ML algorithms. This model allowed for early-stage diagnosis of lung cancer, with remarkable sensitivity and accuracy [188]. Another study highlighted its role on patient surveillance as well as on the detection of mechanisms of resistance in *EGFR*-mutated NSCLC, which was achieved by targeting shorter cfDNA fragments, which enabled the detection of the *T790M* mutation [189]. MRD after surgical resection has also been evaluated in several models combining WGS or ML with genetic and epigenetic features. These models confirmed the excellent performance of circDNA fragment assessment in predicting patient recurrence, which could guide adjuvant treatment decisions and therefore optimize patient management [190,191,192].

Interestingly, fragmentomics enhances the sensitivity of ctDNA-based assays by analyzing cfDNA fragmentation patterns—such as size distribution, end motifs, and nucleosome positioning—that are altered in cancer and detectable even when mutation-based approaches fall below their detection threshold [191,193]. By integrating these multidimensional features, fragmentomics significantly improves early cancer detection, particularly in low tumor burden settings, with clinical studies reporting sensitivity gains from 46% to over 90% at high specificity [194].

As fragmentomics continues to evolve, its applications in liquid biopsy research hold great promise for revolutionizing early cancer detection, monitoring, and treatment decision-making. Future research in this field might further enhance the precision and non-invasiveness of NSCLC diagnostics, improving patient outcomes and personalized care.

## 5. Integrating Emerging Techniques of Translational Research in Oncology

To fully harness the potential of emerging techniques of translational research in oncology, it is essential to recognize their complementary role alongside traditional diagnostic methods. AI, particularly through ML and DL, enhances rather than replaces conventional tools. In radiology, AI improves the sensitivity and specificity of imaging interpretation, aids in the early characterization of pulmonary nodules, and reduces inter-observer variability. In pathology, DL-based algorithms have shown strong performance in subtyping NSCLC, optimizing WSI analysis, and supporting immunohistochemical biomarker quantification, thereby augmenting diagnostic accuracy and reproducibility. Moreover, AI models are increasingly applied in prognostic prediction and treatment response evaluation, offering valuable clinical insights beyond static morphologic assessment.

In parallel, liquid biopsy has emerged as a minimally invasive modality capable of complementing or, in some scenarios, temporally surpassing tissue biopsy. By detecting ctDNA, ctRNA, and other tumor-derived analytes, it allows for dynamic monitoring of disease burden, early assessment of treatment response—often preceding radiologic changes—and the identification of actionable mutations and resistance mechanisms. Importantly, liquid biopsy can capture spatial and temporal tumor heterogeneity, which might be underrepresented in single-site tissue samples. Its role in MRD, assessing molecular relapse, and guiding therapeutic adaptations underscores its expanding clinical value.

Despite these advances, important challenges persist. The generalizability of AI and liquid biopsy technologies is limited by heterogeneity in study designs, variability in analytical platforms, and the absence of universally accepted technical standards. The lack of robust, prospective, multi-center clinical trials further impairs the development of consensus guidelines and regulatory endorsement. Access to these technologies remains uneven across institutions, and both financial and infrastructural barriers restrict their widespread implementation in routine care. For AI in particular, concerns regarding interpretability, model transparency, and validation across diverse populations continue to hinder clinician adoption. Similarly, liquid biopsy faces biological limitations—such as low ctDNA shedding in early-stage disease—and technical constraints in reliably detecting gene fusions or distinguishing tumor-derived alterations from clonal hematopoiesis.

Altogether, while precision medicine in oncology is evolving rapidly—with AI and liquid biopsy at the forefront—it is clear that the field is still in a formative phase. Meaningful clinical integration will require rigorous validation, regulatory harmonization, and equitable access. A multidisciplinary, evidence-driven approach that leverages the strengths of both traditional and emerging modalities will be critical to achieving the promise of truly individualized cancer care. Precision oncology is no longer a theoretical ideal, but its full realization remains a work in progress.

## 6. Conclusions

New advances in translational research have significantly deepened our understanding of tumor biology, particularly the dynamic interplay between cancer cells, the immune system, and the TME. These insights are beginning to translate into clinically actionable strategies that move beyond static biomarkers, toward dynamic, integrative, and biologically informed tools. While AI and liquid biopsy are among the most promising frontiers, the broader translational toolbox—including multi-omics profiling, spatial transcriptomics, and single-cell technologies—is contributing to a paradigm shift in how we approach cancer diagnostics and treatment planning.

From a clinical standpoint, the future of oncology lies in the ability to detect disease earlier, tailor therapies more precisely, and monitor patients more effectively in real time. The success of these innovations will depend not only on technological advancement but on their demonstrable impact on clinical decision-making and patient outcomes. As these techniques continue to evolve and integrate seamlessly into cancer screening and diagnostics, their transformative potential is set to redefine the landscape of healthcare, helping in the discovery of new prognostic and predictive biomarkers and paving the way to more precise and personalized treatments.

## Data Availability

The data that support the findings of this study are available on request from the corresponding author, J.C.B.
